#  Pharmacokinetic profile of sarcin and thionin from *Aspergillus giganteus* and *in vitro* validation against human fungal pathogen

**DOI:** 10.1042/BSR20220229

**Published:** 2022-09-07

**Authors:** Ramya Ravindhiran, Ramya Krishnamurthy, Karthiga Sivarajan, Jothi Nayaki Sekar, Kumarappan Chidambaram, Ali M Alqahtani, Kavitha Dhandapani

**Affiliations:** 1 Department of Biochemistry, Biotechnology and Bioinformatics, Avinashilingam Institute for Home Science and Higher Education for Women, Coimbatore 641 043, Tamil Nadu, India; 2 Department of Pharmacology and Toxicology, School of Pharmacy, King Khalid University, Abha 62529, Saudi Arabia

**Keywords:** ADMET, Antagonism, Aspergillosis, Aspergillus giganteus, GC-MS, Sarcin

## Abstract

Fungal infections are more predominant in agricultural and clinical fields. Aspergillosis caused by *Aspergillus fumigatus* leads to respiratory failure in patients along with various illnesses. Due to the limitation of antifungal therapy and antifungal drugs, there is an emergence to develop efficient antifungal compounds (AFCs) from natural sources to cure and prevent fungal infections. The present study deals with the investigation of the mechanism of the active compounds from *Aspergillus giganteus* against aspergillosis. Primarily, the bioavailability and toxicological properties of antifungal proteins such as, sarcin, thionin, chitinase and their derivatives have proved the efficiency of pharmacokinetic properties of selected compounds. Molecular interactions of selected compounds from *A. giganteus* with the virulence proteins of *A. fumigatus* (UDP-N-acetylglucosamine pyrophosphorylase, N-myristoyl transferase and Chitinase) have exhibited a good glide score and druggable nature of the AFCs. The antagonistic potential of AFCs on the pathogen was confirmed by SEM analysis where the shrunken and damaged spores of AFCs treated pathogen were observed. The integrity of *A. fumigatus* cell membrane and nuclear membrane treated with AFCs were analyzed by determining the release of cellular materials. The effective concentration of AFCs was found to be 250 µg/ml (*P*<0.0001). The GC-MS profiling has revealed the volatile bioactive metabolites present in *A. giganteus*. Further, interaction studies might provide more information on the synergism activity with the non-volatile metabolites which leads to the development of novel drugs for the treatment of aspergillosis.

## Introduction

Emergence of fungal infection has hugely increased in the past three decades worldwide [1]. Fungal pathogens may develop a resistance mechanism against several existing antibiotics [2]. Most of the pathogenic fungi are polymorphic. It can undergo reversible morphological transitions between yeasts in both pseudohyphal and hyphal growth forms [3]. Despite the emergence of invasive fungal infections, therapies mainly rely upon the use of antifungal drugs from synthetic sources namely, Amphotericin B, Azole antifungal compounds (AFCs) and Echinocandins [4]. Most of these drugs are currently used in clinical practices. Unfortunately, the antifungal drugs may interact with other medications and possibly cause side effects, resistance problems, and most of them are fungistatic rather than fungicidal and some of them are often toxic to the patients. These antifungal drugs inhibit a target unique to fungi and they show good therapeutic ratios but their oral bioavailability is considerably low [5,6].

*Aspergillus fumigatus*, aspergillosis causing fungal pathogen, is a life-threatening severe infection provoked by the inhalation of spores and conidia, especially in immunocompromised patients affected by AIDS, organ transplantation or patients who have a long term invasive medical practices and posing a big threat to human health. One kind of alkaloid in *A. fumigatus* represented as ergot alkaloids were found to cause severe health deteriorating problems in both human and animals [6–8]. Apart from causing infections in the host, its pathogenic potential extended towards asthma and leads to other allergic complications in humans and animals. Aspergillus genus was found ubiquitously and have distributed in a wide range of different climatic conditions with pathogenic potential [9]. *Aspergillus fumigatus* cell wall composition relies on the stage of the pathogenic progression in the host; hence, the immune system of the host also get varies on its action [10,11].

Fungi have been considered as an optimal source, producing active metabolites in terms of primary and secondary natural metabolic bioactive components. These compounds are differentiated by their structures and functions [12]. *Aspergillus giganteus* is known to produce small, basic and cysteine-rich antimicrobial compounds that are proved to have maximum antagonistic activity against the number of filamentous fungi. The antifungal protein (AFP) consists of 51 amino acids and it remains inactive in its prosequence form with 91 amino acids that are cleaved by the action of proteases during the secretion process [13]. The exact mechanism of action of AFP is still not explained well. The active metabolite perturbs the plasma membrane of the host cell and subsequently, the synthesis of chitin in filamentous fungi also gets suppressed [14]. In this regard, it might get invading into the pathogenic cell and interact with the nucleic acids of fungi results in the release of nuclear contents [15]. Several studies have shown that AFP and its mechanism of action differed from species to species. It involves either forming a pore on the host cell membrane or inhibit cell wall synthesis and intruding with nucleic acids and their synthesis or inhibiting the protein synthesis thereby interfering with the control of the cell cycle [16].

*In vitro* and *in vivo* studies have proved that *A. giganteus* could be used as a biocontrol agent to inhibit other fungal growth that leads to crop destructions [17–19]. Apart from agricultural purposes, it can be identified as an AFC for the treatment of various fungal infections in humans especially caused by *Candida albicans*, *Aspergillus oryzae*, *Aspergillus niger*, *Fusarium oxysporum* and *Botrytis cinerea* [20–22]. Hence, the present study aimed to investigate the mode of targeted action of AFCs for the inhibition of fungal strains that causes aspergillosis.

## Materials and methods

Based on scientific literatures, we have selected and downloaded the 2D structure of sarcin, thionin, chitinase and their derivatives in .sdf file format. The general description of the selected ligands is tabulated and given as Supplementary File S1.

### Bioavailability and toxicological properties of sarcin, thionin and chitinase and its derivatives of *Aspergillus giganteus*

The ADMET (Absorption, Distribution, Metabolism, Excretion and Toxicological properties) profile of compounds of *A. giganteus* including, sarcin, thionin and chitinase and its derivatives [13,23] were studied by the software pkCSM (http://biosig.unimelb.edu.au/pkcsm/prediction#). Fluconazole is the standard antifungal drug was predicted to compare the efficiency of AFCs of *A. giganteus*.

### Molecular interaction studies

The pathogenic protein targets were downloaded in 3 dimensional, pdb format in the PDB database (http://www.rcsb.org/pdb). The selected proteins namely, UDP-N-acetylglucosamine pyrophosphorylase (6TN3), N-myristoyl transferase (4CAW) and Chitinase (2A3B) are cell wall proteins that are essential for the virulence properties of *A. fumigatus*. Molecular docking studies of compounds with the target proteins were performed using Maestro v11.8 (Schrodinger Release 2018-4: Glide, Schrodinger, LLC, New York, NY, 2018). The downloaded protein structures were prepared by Protein Preparation Wizard module of Schrodinger software.

### Fungal strains and culture conditions

*Aspergillus fumigatus* a pathogenic strain (PSGIMS, Coimbatore) was selected to investigate the mode of action of *A. giganteus* (8408) which was procured from Microbial Type Culture Collection, Institute of Microbial Technology (IMTECH), Chandigarh. The fungal strains were maintained on Czapek yeast extract medium (CYE) at 4°C for storage. Before applications, the fungal strains were revived and subcultured on the same medium at 28 ± 2°C in dark conditions for 4–7 days. Our pilot studies have proved that the active metabolites in the *A. giganteus* is secreted extracellularly [19]. In this regard, the cell-free supernatant was chosen to assess the potential mechanism of AFCs on the pathogenic cell wall.

### Scanning electron microscopic analysis

The cell morphology of the *A. fumigatus* was analyzed by scanning electron microscopy after being treated with the AFCs of *A. giganteus*. The 4-day-old culture of *A. fumigatus* was treated with the AFCs of *A. giganteus* and the inhibition zone was subjected to 4.0% glutaraldehyde in 0.05 mol/l phosphate-buffered saline at 4°C for fixation. The fixation was carried out for 4–6 h, and then the samples were washed with distilled water three times for 20 min each. Sequential dehydration with ethanol dilutions (30%, 50%, 70% and 90% ethanol in distilled water) was performed for 20 min and finally with absolute ethanol for 45 min. Following drying, the samples prepared were mounted on SEM stubs using double-stick adhesive tabs and coated with gold-palladium, and analysed by FE-SEM (Zeiss) instrument by applying 5 kV to view under different resolutions.

### Preparation of antifungal compounds

The cell free supernatant from *A. giganteus* was prepared by the stepwise procedure. Briefly, the fungal strain was grown on the optimized culture medium (CYE) at 28 ± 2°C for 4–7 days. After incubation, the culture was filtered through 8 layered cheesecloth to remove the mycelia. The collected supernatant was filtered through a 0.22 μm membrane filter to obtain cell-free culture filtrates that contains AFCs were stored at 4°C before use. The amount of protein in the cell-free supernatant was determined by Lowry’s method [24].

### Antifungal disruption mechanisms

#### Preparation of mycelial suspension of the pathogenic strain

Inoculum of 5 mm sized mycelial mat with spores of *A. fumigatus* from the freshly grown plate was introduced into the 100 ml of czapek yeast extract broth. It was kept at 28 ± 2°C for 4–7 days in the dark. After incubation, the culture broth was centrifuged at 4000 ***g*** for 20 min to collect the mycelia. The pellet was washed thrice with phosphate buffered saline (pH 7.0). It was resuspended in 100 ml phosphate buffered saline for the following studies.

### Cell membrane integrity on *Aspergillus fumigatus*

#### Cellular leakage of nuclear components via cell membrane

The integrity of the cell membrane of pathogenic strain could be determined by the release of cytoplasmic constituents (DNA and RNA) and cell wall constituents (protein and glucose) [25,26]. The treatment begins with the suspensions of the pathogen were treated with AFCs at various concentrations (50–250 μg/ml) and incubated at 28 ± 2°C under agitation in an environmental incubator shaker for 0, 30, 60 and 120 min. Subsequently, 2 ml of the sample was collected and centrifuged at 12000 ***g*** for 2 min. The supernatant was taken to determine the amount of cytoplasmic constituents such as DNA and RNA by measuring the absorbance at 260 nm.

#### Release of protein and glucose through the cell membrane

The cell membrane integrity of pathogenic strain was evaluated by calculating the release of protein and glucose into the medium. The concentrations of proteins and glucose released from the cell membrane were estimated using Lowry’s method and Anthrone method [24,27]. The treatment was given as same as in the above method (50–250 μg/ml of AFCs and time intervals of 0, 30, 60 and 120 min). About 2 ml of the treated sample was taken in appropriate time intervals (12000 ***g*** for 2 min at 4°C) and read at 670 nm for proteins and 630 nm for glucose.

#### Evaluation of lipid content in the AFCs treated pathogenic cell membrane

Lipids are the major component of the cell membrane to maintain the structure and its integrity [28,29]. The lipid concentrations in the treated pathogenic cell membrane were determined by phosphovanillin method. The 2-day-old mycelia from 50 ml CYE broth were collected by centrifuging it at 4000 ***g*** for 10 min. The pellet was dried with a vacuum freeze drier for 4 h. About 0.1 g of dry mycelia was homogenized with liquid nitrogen followed by the extraction with 4 ml of methanol:chloroform:water mixture (2:1:0.8, v/v/v) in a clean dry test tube with vigorous shaking for 30 min. The tubes were centrifuged at 4000 ***g*** for 10 min. An aliquot of 0.2 ml chloroform and liquid mixture was transferred to another clean dry test tube. To which 0.5 ml H_2_SO_4_ was added and heated for 10 min in a boiling water bath. After that, 3 ml of phosphovanillin reagent was added (vigorous shaking) and kept for incubation at room temperature for 10 min. The amount of lipid content in the pathogenic cell membrane was calculated by measuring the absorbance at 520 nm. Cholesterol can be used as a standard.

### Cell wall protection mechanisms

#### Sorbitol assay

The effect of the AFCs on the pathogenic cell wall could be determined by with and without the addition of sorbitol in the medium. Sorbitol act as a fungal cell wall osmotic protective agent, when this sorbitol is added to the culture medium it protects the cell wall of fungi [30]. This sorbitol assay implicated the cell wall as one of the possible cell targets for the product tested thereby used to evaluate the possible mechanisms of AFCs on the pathogenic cell wall. The sorbitol was added to the culture medium to give a final concentration of 0.8 M. Then the plate was incubated at 25°C. The plates were read after 48 h and 7 days at 595 nm.

#### Measurement of extracellular pH changes in treated *Aspergillus giganteus*

In order to check the cell wall permeability, the pH of the medium could be monitored by pH meter. The experiment started with the inoculation of pathogenic fungal suspensions into the CYE medium and kept for incubation in a moist chamber at 28 ± 2°C for 2 days. Then it was centrifuged at 4000 ***g*** for 20 min and the pellet was washed for 2–3 times with sterile double distilled water. Then different concentrations of AFCs (50–250 μg/ml) were added and the pH of the medium was check for every 0, 30, 60 and 120 min to observe the changes in the pH in the treated sample.

#### Evaluation of cytotoxicity of AFCs by MTT assay

Cytotoxicity of the AFCs present in the culture filtrates of *A. giganteus* were determined using MTT (3-(4,5-dimethylthiazol-2-yl)-2,5 diphenyltetrazolium bromide) assay. The cell viability was measured using the formation of formazan product from MTT upon the treatment with the AFCs at various concentrations. Initially, the whole blood cells were seeded in a 96-well plate and the AFCs (50–250 μg/ml) were added to the appropriate treatment wells. The plates were allowed to incubate at 37°C in a humidified CO_2_/95% air mixture for 24 h. The MTT were added to each well and incubate for 3 h and observed for the formation of formazan crystals. Dimethyl sulfoxide (Himedia) was added to each well for the complete dissolution of formazan crystals. MTT and lymphocytes cells served as negative control. Blank was prepared by adding MTT and PBS (phosphate-buffered saline) in the well. Finally, the plate was measured at 570 nm in ELISA reader.

#### Gas chromatography-mass spectrometry (GC-MS) profiling of the bioactive metabolites of *Aspergillus giganteus*

Besides, the non-volatile AFCs in *A. giganteus* volatile compounds responsible for the antagonistic potential was studied by GC-MS analysis. The bioactive metabolite profile of *A. giganteus* was studied by procuring the culture filtrates from the *A. giganteus* and subjecting them to ethyl acetate extraction (1:1 v/v). The collected extract was concentrated in a rotary evaporator and 1 μl of this sample was subjected to GCMS analysis in Thermo GC-MS DSQ instrument. A standard non-polar column was used with helium as carrier gas (flow rate 1ml/min) and oven temperature set from 70 to 260°C. The metabolites obtained in the spectra were compared to the reference spectra in the NIST- Wiley database.

### Statistical analysis

All the experiments were carried out in triplicates and the values are expressed as mean ± SD. The mechanistic studies were analyzed using two-way ANOVA in order to determine the statistical significance using the statistical package in the GraphPad Prism 9.3.0 version. The difference is significant for *P*-value ≤0.05 of the performed experimental data.

## Results

### Pharmacokinetic profile of sarcin, thionin, chitinase and its derivatives of *A. giganteus*

The ADME property of the fluconazole, sarcin, thionin, chitinase and its derivatives is listed in Table 1. All the selected compounds were found to have a good absorption level except the chitinase derivative. The highest absorption was found with sarcin, thionin and their derivatives. The intestinal absorption and skin permeability profile represent that the selected compounds have a good absorption profile compared with the standard drug, fluconazole. Sarcin from *A. giganteus* is found to have been distributed into the blood–brain barrier and central nervous system. The compounds were observed as neither substrate nor inhibitor for the cytochrome P450, which is an important detoxification enzyme found in the liver. The studies have given the way to conclude that the sarcin and its derivatives of the *A. giganteus* have shown a good ADME profile while comparing with the standard antifungal drug. The toxicological properties of the sarcin, thionin, chitinase and its derivatives showed fewer toxic properties and it is contemplating that the selected compounds are safe for use to treat the fungal infection (Table 2).

**Table 1:** ADME properties of sarcin, thionin, chitinase and its derivatives of *Aspergillus giganteus*

PubChem ID	Absorption				Distribution		Metabolism				Excretion	
	Water solubility (log mol/L)	Intestinal Absorption (human) (% absorbed)	Skin permeability (logKp)	Oral absorption	BBB permeability (log BB)	CNS permeability (log PS)	CYP2D6 substrate	CYP3A4 substrate	CYP2D6 inhibitor	CYP3A4 Inhibitor	Total clearance (log ml/min/kg	Renal OCT2 substrate
3365	-3.293	94.964	-2.8	High	-1.067	-3.185	No	No	No	No	0.29	No
3032391	-3.027	100	-2.735	High	-1.105	-3.779	No	No	No	No	0.609	No
65044	-3.487	91.621	-3.31	High	-0.158	-1.952	No	No	No	No	0.153	Yes
462371	-2.071	74.632	-3.157	High	-0.157	-0.157	No	No	Yes	No	1.175	Yes
122678533	-2.486	93.484	-2.741	Low	0.17	-2.812	No	No	No	Yes	0.075	No
88094842	-5.708	93.533	-1.499	High	0.703	-2.125	No	Yes	No	No	0.14	No
86223064	-3.391	95.19	-2.765	High	-0.457	-2.659	No	Yes	No	Yes	0.914	Yes
86223063	-3.208	94.181	-2.766	High	-0.274	-2.554	No	Yes	No	No	1.04	Yes
6857375	-1.38	31.963	-3.234	Medium	-0.618	-3.694	No	No	No	No	0.711	No

**Table 2:** Toxicological profile of sarcin, thionin, chitinase and its derivatives of *Aspergillus giganteus*

Ligand	AMES toxicity	Max. Tolerated dose (human) (log mg/kg/day)	HERG inhibitor		Oral acute toxicity (LD50) (mol/kg)	Oral rat chronic toxicity (LOAEL) (Log mg/kg bw/day)	Hepatotoxicity	Skin senitization	*T.pyriformis* toxicity (log µg/L)	Minnow toxicity (log mM)
			I	II						
3365	No	0.114	No	No	2.328	1.033	Yes	No	0.312	3.872
3032391	Yes	0.649	No	No	2.454	0.855	Yes	No	0.285	2.272
65044	Yes	-0.3	No	No	2.532	1.885	No	No	1.041	0.686
462371	Yes	-0.188	No	No	2.478	1.661	No	No	0.495	1.142
122675833	Yes	0.942	No	Yes	2.771	0.207	Yes	No	0.285	0.502
88094842	No	0.18	No	No	2.137	0.794	No	Yes	3.193	0.494
86223064	No	-0.21	No	Yes	2.588	0.663	Yes	No	0.297	-1.049
806223063	No	-0.187	No	Yes	2.541	0.778	Yes	No	0.297	-2.017
6857375	No	1.944	No	No	1.547	3.406	No	No	0.285	4.705

### Molecular interaction of bioactive compounds with the virulence proteins

**Figure 1:**
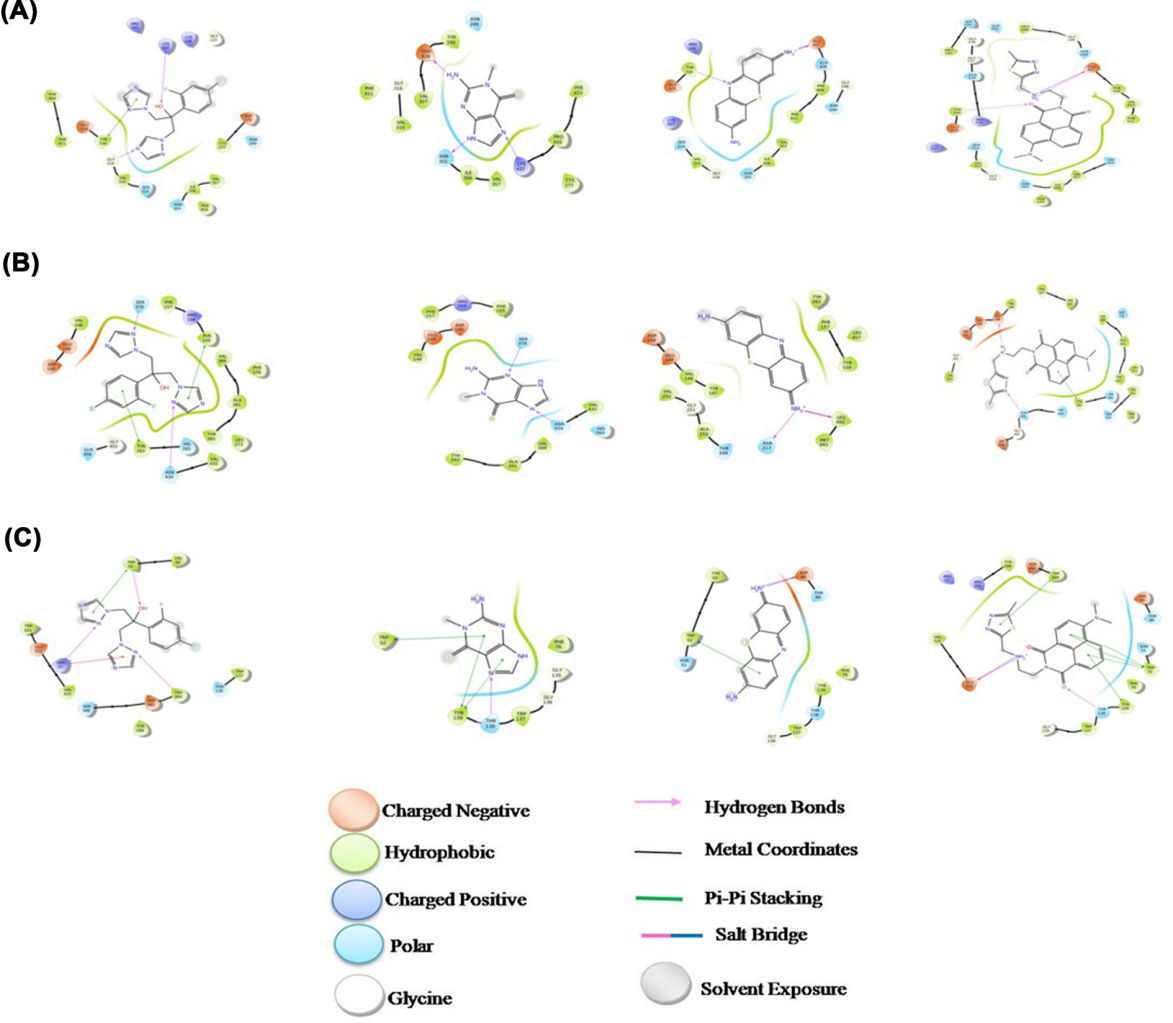
Molecular interactions of Fluconazole (3365) and Sarcin (3032391 and 65044) from *A. giganteus* with the various pathogenic target proteins of *A. fumigatus* (**A**) Antagonistic compounds with *Aspergillus fumigatus* target protein, 6TN3. (**B**) Antagonistic compounds with *Aspergillus fumigatus* target protein, 4CAW. (**C**) Antagonistic compounds with *Aspergillus fumigatus* target protein, 2A3B.

Molecular simulation studies were performed to identify the best interaction of ligand molecules with the pathogenic target proteins which leads to the development of a wide range of drugs after several layers of screening. The best selected three ligand molecules with the selected target proteins were docked at its active site. The missing hydrogen atoms were incorporated and proper ionization states were generated using the OPLS-2005 force field. For optimal molecular interactions, the minimization was done at last until the heavy atoms are converged to an RMSD of 0.30 Å. A grid box was generated around the co-crystallized ligand in the receptor protein that allows the molecular docking into the active site of the protein. The cubic boxes have been created with the coordinates in the receptor grid generation correspond to the 3.336, 15.597 and 4.868 in *x*, *y* and *z* directions respectively, centered on the centroid of the co-crystallized ligand. The weak force, Van der Waals radius scaling factor was set to 1.0 Å and the partial charge of cut off was maintained at 0.25 with no constraints. The sarcin, thionin, chitinase from *A. giganteus* and fluconazole was docked with the virulence proteins of pathogenic *A. fumigatus*. Table 3 shows the molecular interactions of sarcin, thionin, chitinase and fluconazole with the pathogenic target proteins. The docking images with the virulence proteins are depicted in Figure 1.

**Table 3:** Molecular Interaction of sarcin (3032391), thionin (462371), chitinase (86223064) from *A. giganteus* and Fluconazole with the virulence proteins for aspergillosis

Molecular interaction	6TN3- *A. fumigatus*				4CAW- *A. fumigatus*				2A3B- *A. fumigatus*			
	3365	3032391	462371	86223064	3365	3032391	462371	86223064	3365	3032391	462371	86223064
Docking score	-5.225	-6.248	-5.308	-4.688	-6.408	-6.5	-7.623	-7.006	-3.976	-5.565	-6.75	-7.397
Glide energy	-37.578	-31.16	-33.495	-29.053	-39.89	-29.986	-61.952	-51.311	-35.651	-25.489	-29.008	-49.902
No. of hydrogen bonds	2	3	2	2	2	2	2	2	3	1	1	2
Interacting residues	LYS 437 GLY 316	GLU 329 ASN 355 LYS 437	TYR 330 GLU 407	GLU 407 TYR 330	ASN 434 SER 378	SER 378 ASN 434	ASN 213 LEU 492	GLU 149 GLN 456	TRP 52 ARG 59 TRP 384	THR 138	ASP 90	THR 138 GLU 322
Glide h bond	0	0	0	0	0	0	0	0	0	0	-0.2	-0.015

### Scanning electron microscopic analysis

**Figure 2:**
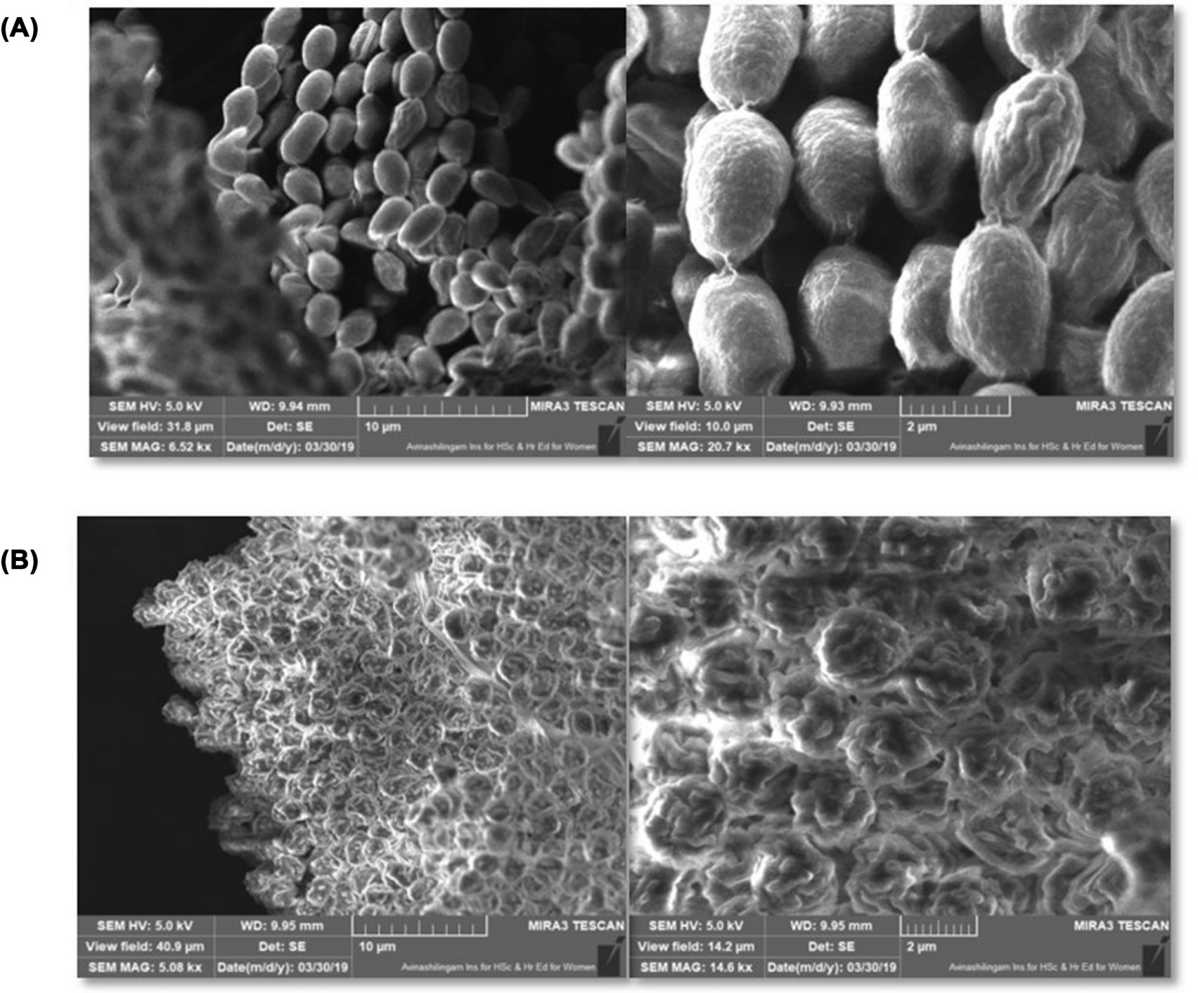
Scanning electron microscopic images of control and AFCs treated pathogen (**A**) Control in different resolutions. (**B**) AFCs treated *A. fumigatus*.

Morphological changes of pathogenic fungi treated with antagonistic fungi were well established by scanning electron microscopy (Figure 2A,B). Extensive damage and collapsed structure with hyphal distortion of *A. fumigatus* was noticed in plates treated with AFCs of *A. giganteus*. In control plates, regular-shaped homogenous hyphae were observed and it was confirmed under different resolutions.

### Antifungal mechanisms

The cell-free supernatant was prepared to examine the mechanism of action of AFCs on the pathogenic cell wall and cell membrane. The AFCs are extracellular and protein in nature. The protein concentrations were estimated in the AFC preparations and were found to be 0.75 mg/ml.

### Cell membrane integrity assay of AFCs on pathogenic cell

Cell membrane integrity assay was performed to investigate the mechanistic action of AFCs in the culture filtrates of *A. giganteus* on the pathogenic cell membrane, *A. fumigatus*.

### Release of nucleic acids

**Figure 3:**
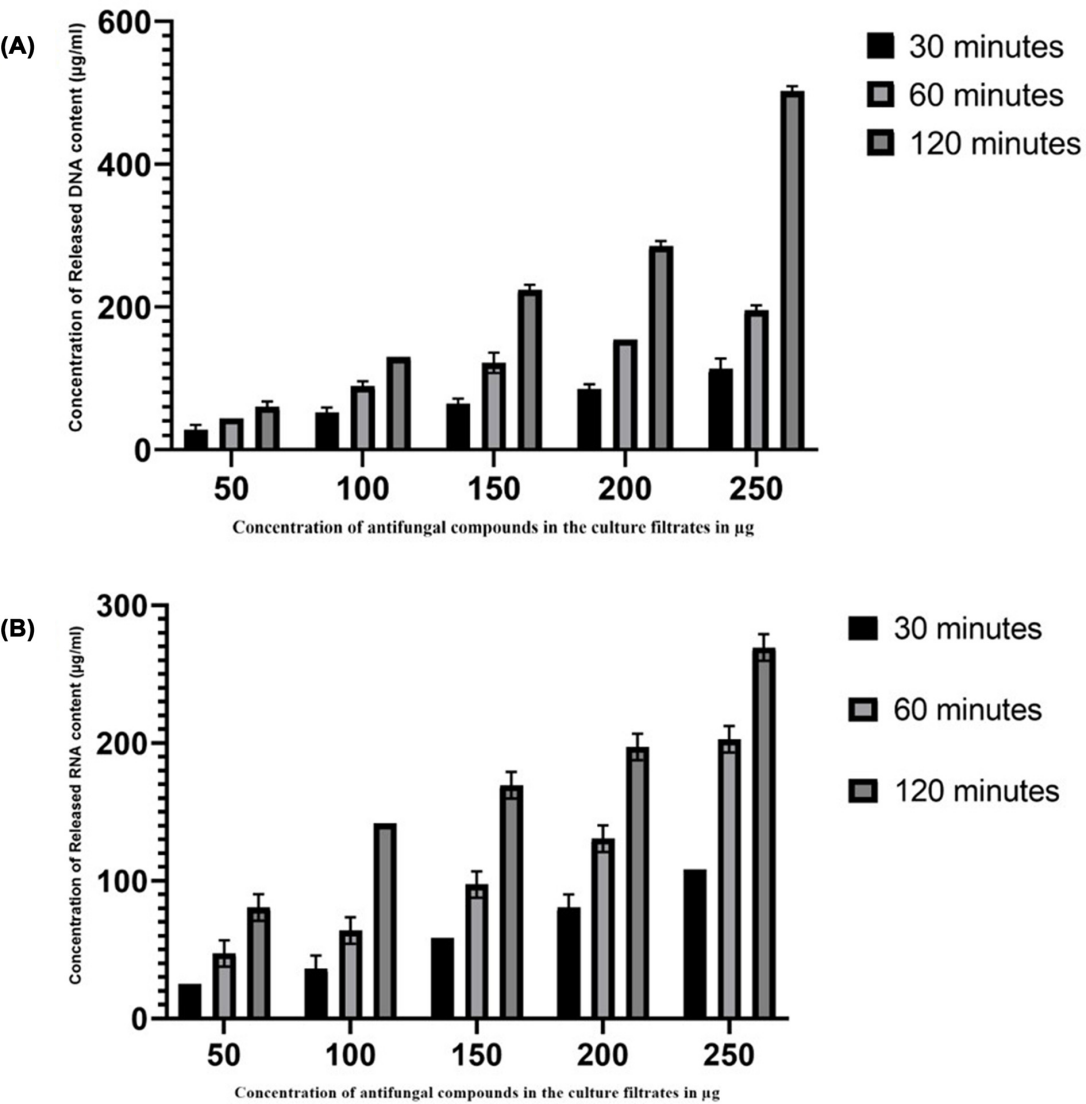
Release of nucleic acids from cell membrane of *Aspergillus fumigatus* treated with different concentrations of AFCs of *Aspergillus giganteus* (**A**) Leakage of DNA from AFCs treated pathogen. (**B**) Leakage of RNA from AFCs treated pathogen. The results are expressed as µg/ml of components released. Data are represented as mean ± SD (*n*=3) *P*<0.001.

The released materials such as DNA and RNA were used to analyse the effects of AFCs on the pathogenic cell membrane by measuring the absorbance at 260 nm. The released DNA content by the action of AFCs was depicted in Figure 3A and the release of DNA into the medium was observed maximum at 120 min of exposure of AFCs on pathogenic cellular membrane. The OD_260_ values were noted as 0.05 ± 0.01, 0.10 ± 0.01, 0.18 ± 0.02, 0.22 ± 0.02 and 0.40 ± 0.03 for the concentration of AFCs 50, 100, 150, 200 and 250 µg, respectively (*P*<0.0001). As clearly stated, increased concentration of AFCs in *A. giganteus* have disturbed the cellular membrane of *A. fumigatus*, thus the genetic material is released into the medium.

AFCs in the culture filtrates also targets RNA, another important genetic material. Figure 3B shows the results of cell membrane integrity assay for the release of RNA components. Maximum leakage of RNA components was noted at 120 min of treated pathogenic strain with the concentration of AFCs with the range of 50, 100, 150, 200 and 250 µg, and the amount of released constituents was observed as 75 ± 0.01, 141.6 ± 0.01, 158.3 ± 0.02, 198.7 ± 0.02 and 258.3 ± 0.01 µg/ml, respectively (*P*<0.0001). The AFCs in the culture filtrates of *A. giganteus* proved to be an effective tool to disrupt and damage the pathogenic fungal cell membrane.

### Leakage of protein and glucose

**Figure 4:**
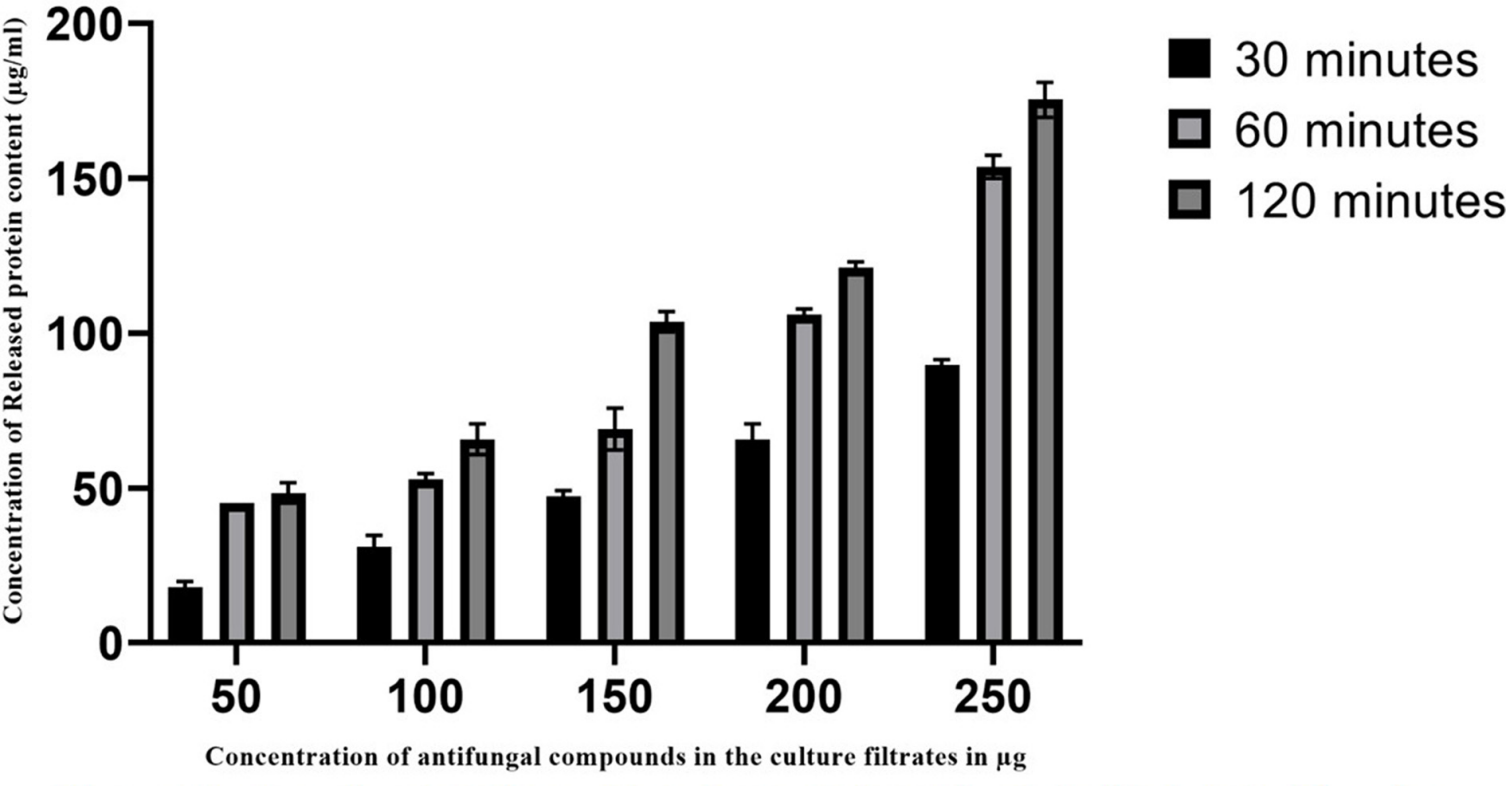
Leakage of proteins from cell membrane of *Aspergillus fumigatus* treated with various concentrations of AFCs The results are expressed as µg/ml of proteins released. Data are represented as mean ± SD (*n*=3); *P*<0.001.

**Figure 5:**
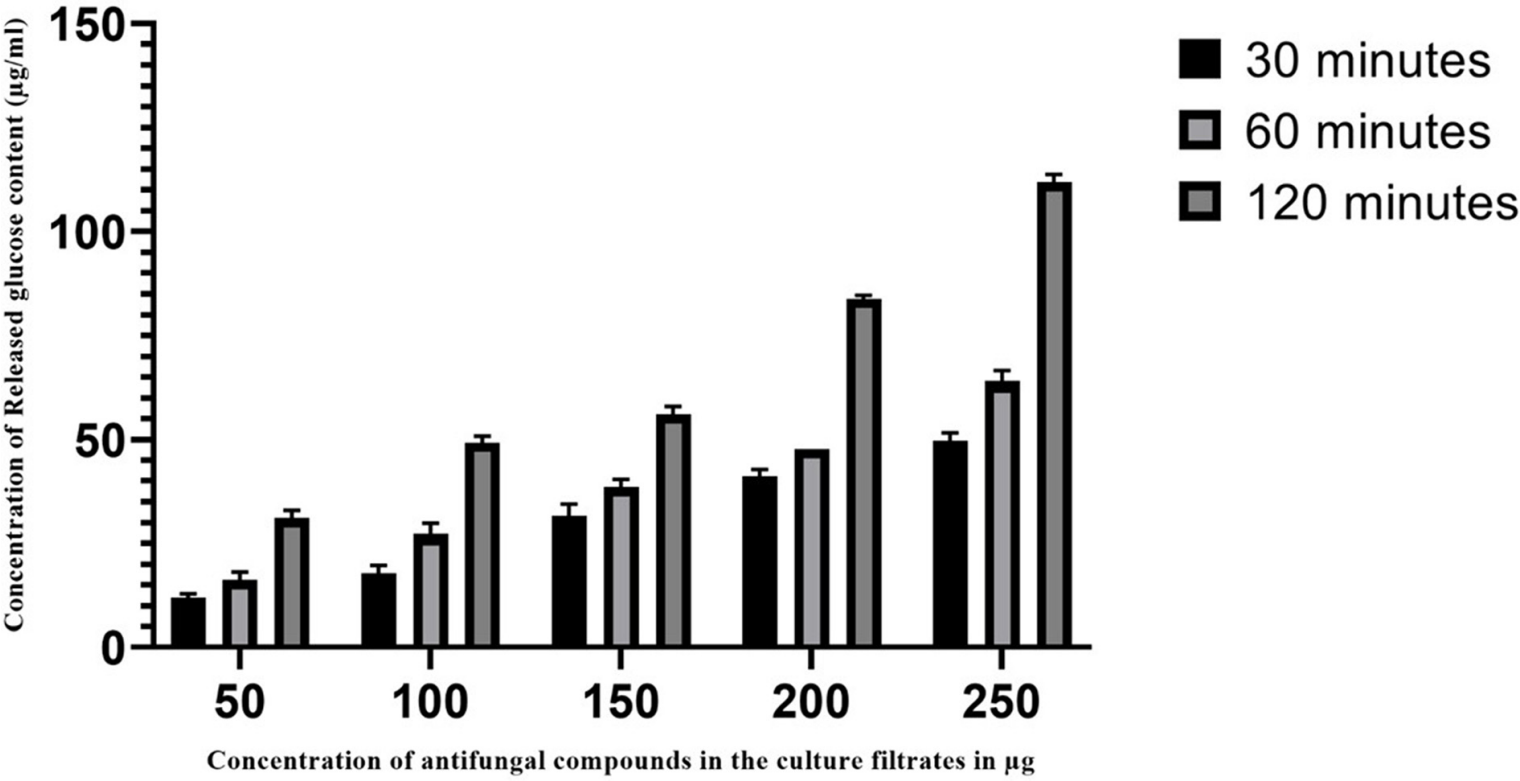
Leakage of glucose from cell membrane of *Aspergillus fumigatus* treated with various concentrations of AFCs The results are expressed as µg/ml of glucose released. Data are represented as mean ± SD (*n*=3); *P*<0.001.

The amount of released protein and glucose were measured by their absorbance at 670 and 630 nm, respectively. Figures 4 and 5 shows the action of AFCs on the pathogenic cell membrane by estimating the released protein and glucose contents. The release of cellular compounds in the treated groups has increased in a concentration- and time-dependent manner.

The enhancement of released protein contents was observed in the treated *A. fumigatus* with AFCs (50, 100, 150, 200 and 250 µg/ml). Indeed, the released contents were found to be maximum at 60 min (*P*<0.0001) and 120 min (*P*<0.0001) of treatment with AFCs which reflects that AFCs in *A. giganteus* has potential effect on the pathogen and causes damage to the cell membrane thus, releasing the protein contents within a short period.

Leakage of glucose in the treated sample was found to be increased with exposure to increased concentration of AFCs with increased time. Release of increased glucose contents were observed at OD_530_ with exposure of AFCs to *A. fumigatus* at 120 min of treatment (*P*<0.0001). The amount of glucose leaked by the AFCs (50, 100, 150, 200 and 250 µg/ml) at 120 minutes treated sample was 30.6 ± 0.01, 47.6 ± 0.01, 53.8 ± 0.01, 82.68 ± 0.01 and 109.8±0.01 µg/ml. The leakage of components (DNA, RNA, protein and glucose) were released into the medium at a higher level in the treated pathogen than that of control (without AFCs treatment).

### Release of lipids during membrane distortion

**Figure 6:**
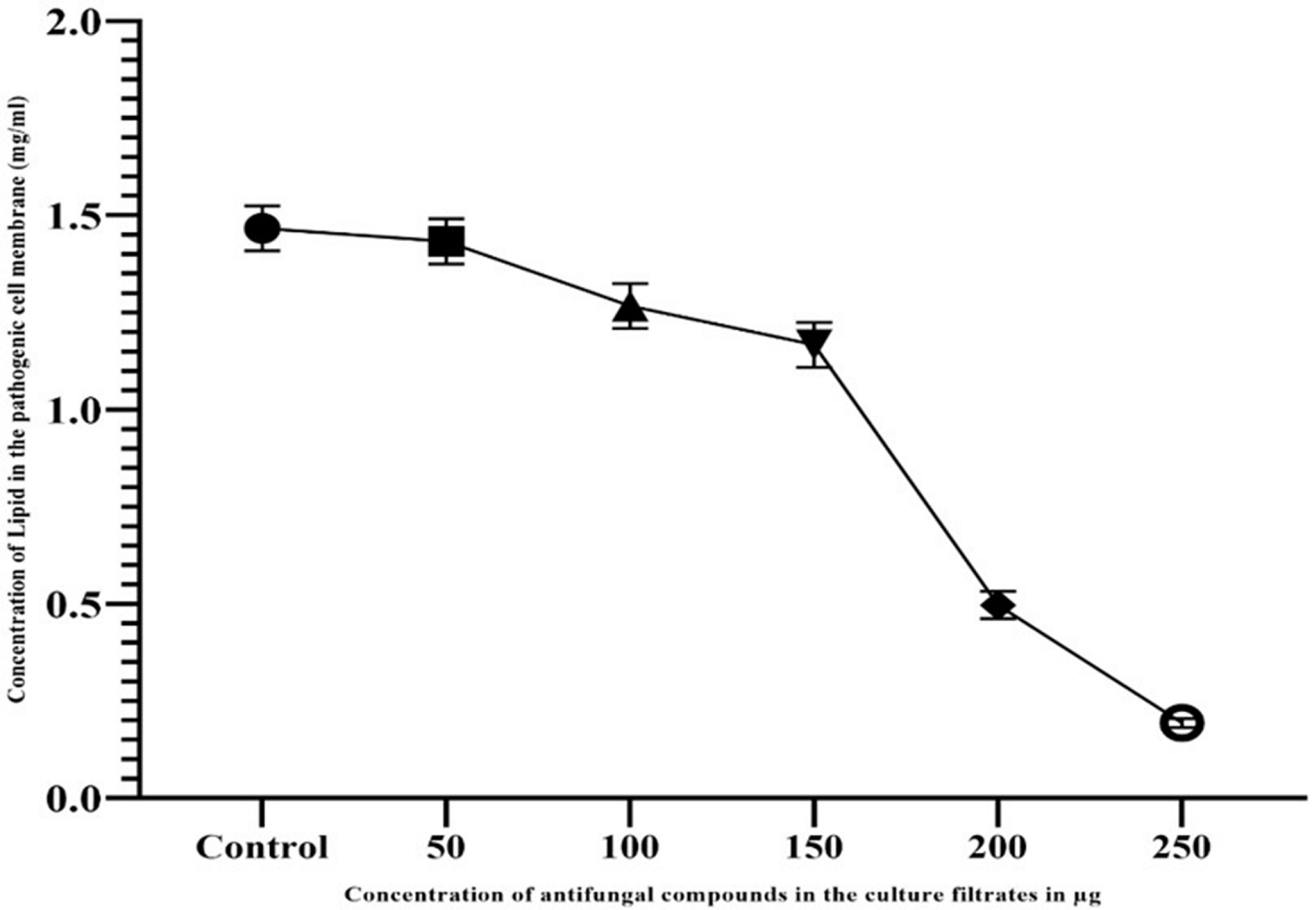
Evaluation of lipid contents in the *Aspergillus fumigatus* cell membrane treated with different concentrations of AFCs Data are expressed as mean ± SD (*n*=3); *P*<0.001.

The lipid content in the treated pathogenic cell membrane was measured by the absorbance at 520 nm. Results of decreased lipid contents in AFCs treated *A. fumigatus* are represented in Figure 6. As depicted in the graph, the AFCs in the culture filtrate of *A. giganteus* have an inverse proportion of the lipid contents in *A. fumigatus* cell membrane. The amount of lipid contents for the increased concentration of AFCs in *A. giganteus* was analyzed using phosphovanillin method. The lipid contents in *A. fumigatus* cell membrane were found to be 0.5 ± 0.03 and 0.2 ± 0.02 mg/ml for 200 and 250 µg/ml of AFCs, respectively (*P*<0.0001).

### Cell wall protection assay

#### Sorbitol assay

For elucidating the action of AFCs in *A. giganteus*, the sorbitol assay was performed on the pathogenic fungal cell wall with and without sorbitol in the medium. Since sorbitol is acting as a cell wall protectant, the pathogenic strain was observed to be intact in the medium inoculated with 0.8 M sorbitol even in the presence of AFCs at different concentrations (Table 4). Thus the AFCs could not damage the cell wall in the presence of sorbitol indicating its mode of action.

**Table 4:** Effect of AFCs of *Aspergillus giganteus* on the cell wall of *Aspergillus fumigatus* in the presence and absence of sorbitol

S.No	Concentration of AFCs (µg/ml)	Medium with 0.8 M sorbitol	Medium without 0.8 M sorbitol
1.	Control (without AFCs)	-	-
2.	50	-	-
3.	100	-	-
4.	150	-	+
5.	200	-	+
6.	250	-	+

(+) Inhibition (-) No inhibition.

#### Extracellular pH in AFCs treated *Aspergillus fumigatus*

**Figure 7:**
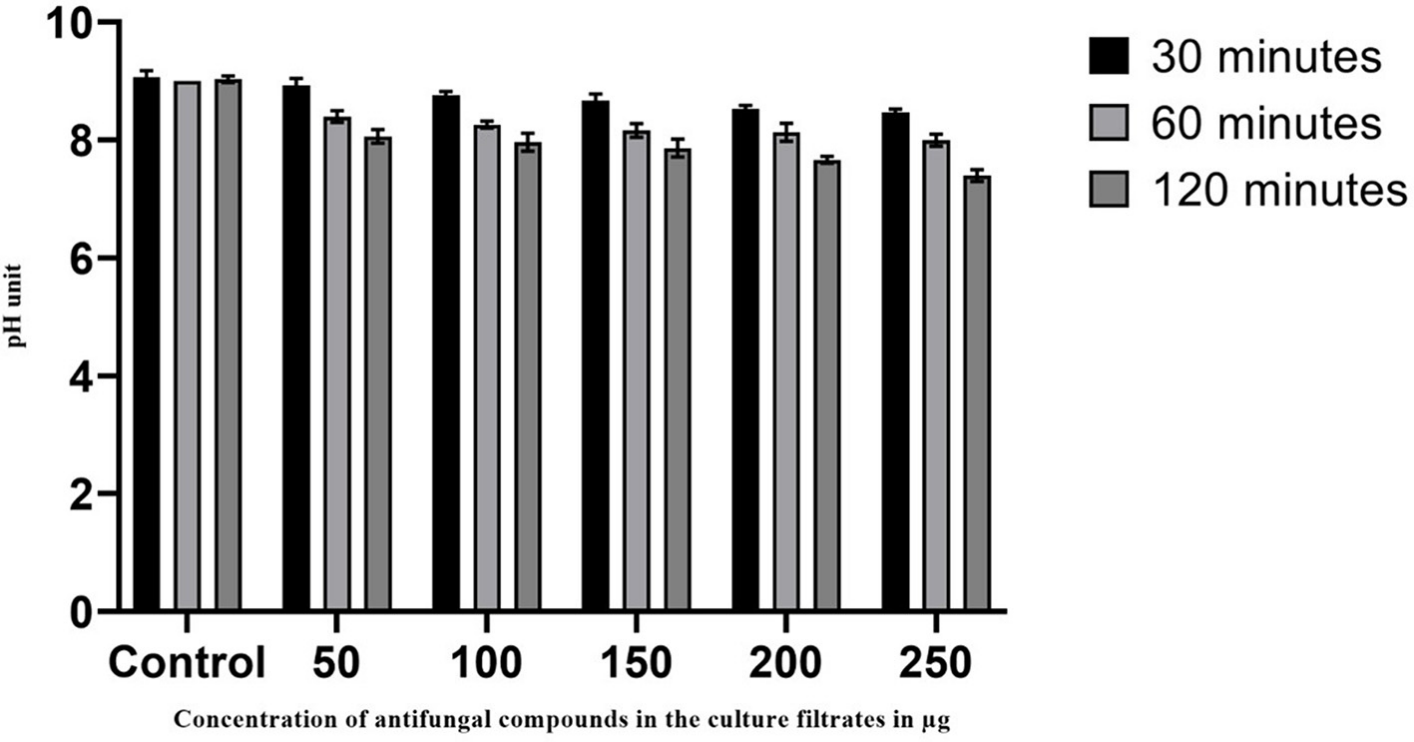
Determination of pH changes observed with different concentrations of AFCs on *Aspergillus fumigatus* The notable changes in the pH were observed. Data are expressed as mean ± SD (*n*=3); *P*<0.001.

Examination of pH changes observed in the AFCs treated pathogenic strain was conducted using a pH meter. Results of pH variation are represented in Figure 7, where pH was shown to decrease gradually with time exposure and AFCs concentration. The pH level began to fall after 30 min of treatment compared with that of control (without AFCs). The decreased pH level in the treated pathogenic cell has revealed that might be some acids or any other acidic metabolites in the pathogenic strain may be leaked into the medium, thus changes in the pH were observed.

#### Cell viability of antifungal compounds by MTT assay

**Figure 8:**
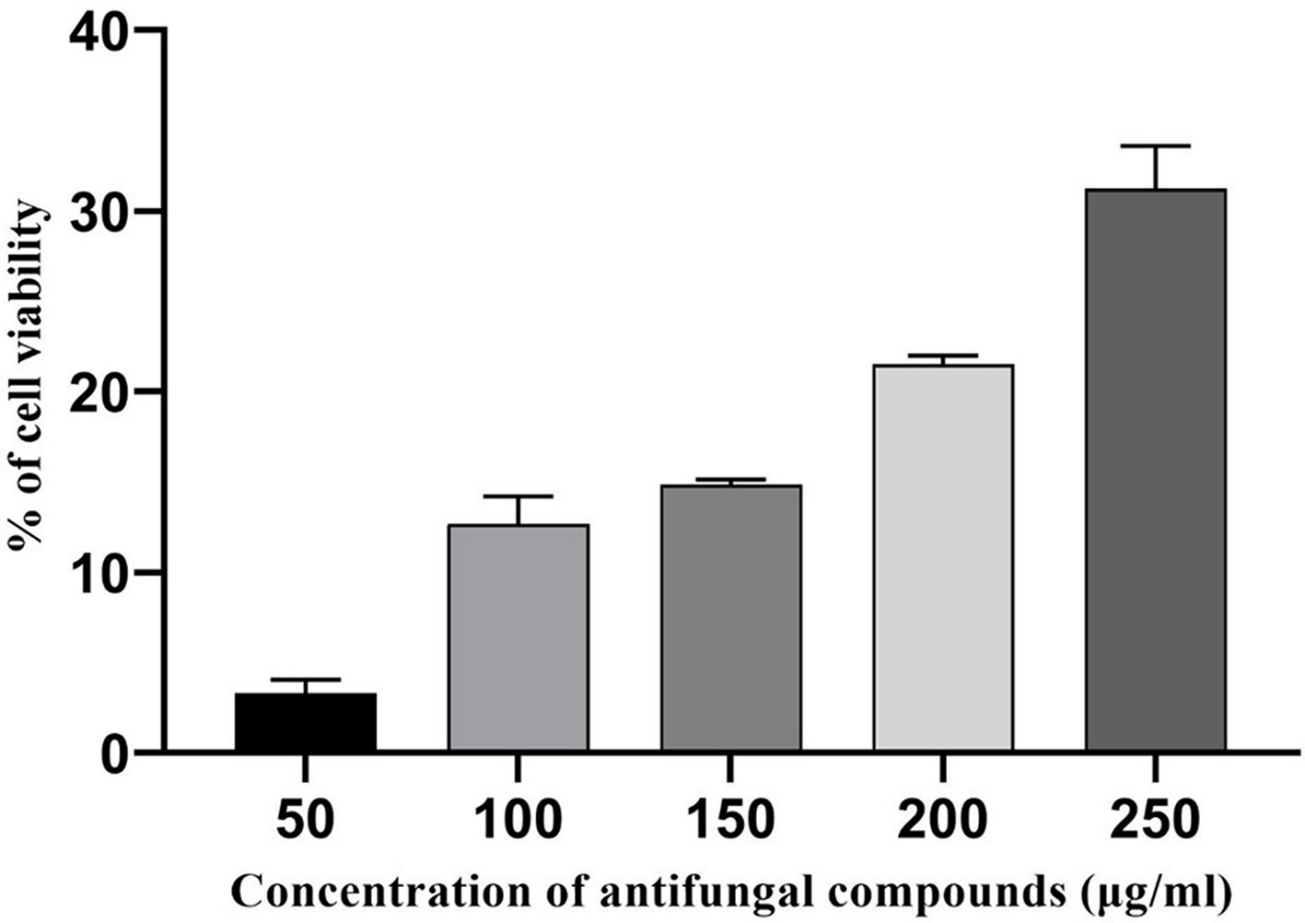
Cytotoxic effects of AFCs of *Aspergillus giganteus* by MTT assay Results are expressed in mean ± SD (*n*=3); *P*<0.001.

The yellow colored MTT compound is reduced to purple formazan by the action of mitochondrial enzymes of viable cells (Whole Blood Cells). The cytotoxic effects of AFCs (50–250 μg/ml) on lymphocyte cells were observed as the antiproliferative effects against the lymphocytes when treated for 24 h. Cells were noted for the gradual decrease in number compared with the control group. The AFCs were found to significantly suppress the proliferation of lymphocytes at even at 200 µg/ml (*P*<0.0001) concentration (Figure 8); thus, it represents the least toxic level to the lymphocytes. From the results, it was clear that the AFCs of *A. giganteus* are found to be safe.

#### GC-MS profile of the bioactive metabolites

**Figure 9:**
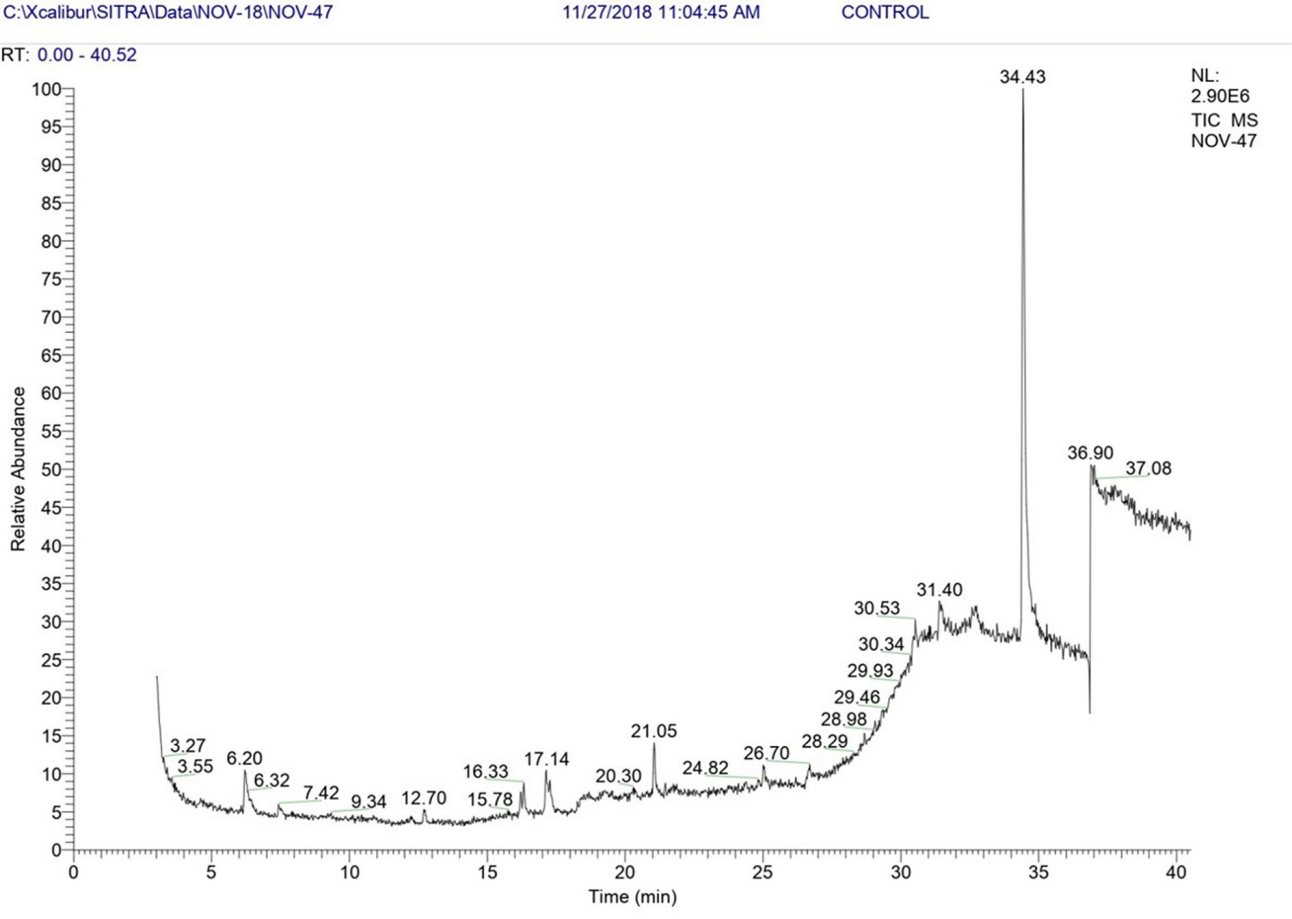
GC-MS chromatogram of culture filtrates of *Aspergillus giganteus*

The ethyl acetate extract of the culture filtrates of *A. giganteus* were subjected to GC-MS analysis and the run resulted in a GCMS chromatogram with their relative abundance. Approximately 30 compounds in the spectrum obtained (Figure 9) were identified as fatty acids, methylated esters of fatty acids, flavones and sterol intermediates from polyketide synthase pathway. Among these compounds, the peaks of relatively abundant compounds were analysed and the compounds are listed in Table 5. The list of fatty acids and methyl esters identified are also presented in Supplementary File S2. Several fatty acid metabolites, have been identified as AFCs which are biodegradable with high specificity for many pathogenic fungi. It has also been found that pathogenic fungi do not become resistant with constant exposure to these antifungal fatty acids. These fatty acids incorporate themselves into the cell membranes of target pathogens and increase membrane fluidity and disruptions in the arrangement of membrane proteins which eventually releases the cellular constituents.

**Table 5:** Bioactive compounds identified in the GC-MS spectrum of *Aspergillus giganteus*

S. No	Retention time	Compound name	Approximate relative abundance (%)
1	15.53	6- hydroxyl-4- (methylthio) indane	10–12
2	21.05	1,2,4- Trioxolane-2- octanoic acid, 5- octyl- methyl ester	15
3	24.82	Cyclopropane butanoic acid	13.5
4	30.49	Lucenin 2	27
5	31.41	5,7,9 (11)- Androstatriene, 3- hydroxyl-17- oxo	35
6	34.46	13- Docosenamide, (Z)	100
7	38.13	Fenretinide	33.5

## Discussion

The treatment and discovery of suitable drugs for fungal infections still pose formidable challenges in the world [31,2]. The application of antimicrobial compounds from natural sources acts as good platform for the drug development [32]. The screening of effective antibiotics from natural sources had been highlighted in the past. Nowadays, the ratio of discovering new and valuable antibiotics falls annually; hence, there is a need for the production of novel antibiotics for the treatment of infections. Researchers and scientists have focused to discover antimicrobial compounds from antagonistic microorganisms [33,34].

Several studies claimed that the AFCs in *A. giganteus* are protein in nature [35–37]. *Aspergillus fumigatus* a well known aspergillosis causing organism was targeted by the antagonistic fungus to inhibit its pathogenesis. Indeed, the exact underlying mode of action of *A. giganteus* remains elusive. Nevertheless, it is popular for producing a kind of AFC and AFP [12].

The *in silico* approaches have provided a way to understand the nature and characteristics of the molecules in the candidate to develop a novel drug by pharmaceutical industries. Based on the literature studies the sarcin, thionin, chitinase and its derivatives from the *A. giganteus* and their structures were downloaded from the PubChem databases. Their ADMET (Absorption, Distribution, Metabolism, Excretion and Toxicity) profile was predicted by the online software. The pathogenic target proteins responsible or the virulence nature is downloaded from the PDB databases. The molecular interaction studies have proved the active binding of ligand molecules with the target proteins of *A. fumigatus*; thus, it can be used to prevent and manage of aspergillosis. The present study confirmed that the AFCs from *A. giganteus* have proved their druggable nature and is an effective lead compound for pharmaceutical industry to develop novel drugs.

AFCs caused damage to the structure of pathogenic strain, *A. fumigatus* and it was confirmed by SEM analysis. The structural distortion of treated pathogenic strain has proved the antagonistic nature of AFCs of *A. giganteus*. Several research studies have correlated with the results of our present study [38–40].

The mechanism of *A. giganteus* AFCs against *A. fumigatus* has been demonstrated. Many studies have supported the techniques used to determine the mode of action of antagonistic compounds [28,37]. Indeed, the AFCs are extracellular and protein in nature, the cell free supernatant of *A. giganteus* was prepared to study its mechanisms. Different concentrations of AFCs in culture filtrates such as 50, 100, 150, 200 and 250 µg/ml was selected for testing its potential on the pathogenic cell membrane and cell wall.

In the present study, the mechanistic action of AFCs of *A. giganteus* was determined by assessing the integrity of the cell membrane and cell wall of the pathogenic strain, *A. fumigatus*. The fungal cell wall and cell membrane are essential for maintaining the structure and function of fungi [33,34,41]. The action of AFCs from *A. giganteus* was proved using the released cellular contents through pathogenic cell membrane into the medium. The release of cell constituents in the treated pathogenic strain was measured using absorbance 260 nm for nucleic acids, 670 nm for proteins and 630 nm for glucose. In this context, the cellular constituents were released into the medium in a time- and concentration-dependent manner. The cell membrane provides a barrier for transporting extra and intra cellular constituents [25,42]. The proteins and glucose are the important cell membrane components needed for the maintenance of cell membrane integrity and function. Cytoplasmic constituents such as DNA, RNA, protein and glucose that are released from the cell membrane of pathogenic strain to the medium is considered as the indicator for the disturbance and damage occurred in the membrane integrity by the AFCs used. The activity of AFCs on pathogenic fungal cell membrane, clearly stated that the released constituents such as DNA and RNA were released gradually into the medium with the increased time of exposure and concentration of AFCs.

Lipid is the important component in the cell membrane responsible for maintaining the permeability, fluidity and function of integral membrane proteins. Several AFCs have already been reported that the main target for affecting the pathogenic fungi is lipids and mechanism behind is either inhibiting the lipid biosynthesis or binding to it, thus causing formation of pores in the cell membrane [29]. The present study represented that the AFCs of *A. giganteus* has effectively exhibited their potential on the lipids in the cell membrane of *A. fumigatus*. The increased exposure of AFCs has a great effect on the cell membrane, thus reducing the lipid contents in the pathogenic cellular membrane. Several scientific studies have mentioned the mechanisms used to study the cell membrane integrity and have proved the damage of cellular membrane occurred with treated antimicrobial substances leading to the release of macromolecules [43–45].

Fungal cell walls are dynamic, very complex structures and they are responsible for maintaining the shape and the integrity of the fungal cell [46]. Sorbitol is an osmotic protectant that protects and stabilizes the fungal cell wall, thus protecting the fungi from environmental stress especially during osmotic changes. Our results are encouraging that the medium without sorbitol showed the inhibition with different concentrations of AFCs. Medium with sorbitol acts as a cell wall protectant, therefore, the pathogenic cells remain undamaged in the presence of AFCs. Similar results were observed with various studies [28,47]. Therefore, the sorbitol assay reiterated that the AFCs act on the cell wall of pathogenic *A. fumigatus*.

The transport of ions and their permeability via cell membrane is managed by the structure and composition of cell wall and cell membrane. Any disturbance in the ion homeostasis ultimately affects the cell metabolism and leads to the death of pathogenic strains [48]. Generally, AFCs affect the lipids in the pathogenic cell membrane, thus disturbing the structure of cell membrane and it might become more permeable causing release of some ions and acidic metabolites into the medium where changes in the extracellular pH were noted. Moreover, our results also explained that the extracellular pH of the treated *A. fumigatus* was observed with increased exposure of time and AFCs concentrations.

Cell viability of antimicrobial substances was measured by microculture tetrazolium (MTT assay) [49,50] assay. The cell viability on peripheral lymphocyte cells is used to determine the toxicity of antimicrobials. This method is an effective, reproducible and cheapest method to evaluate the toxicity of antimicrobial compounds, minimizing the animal sacrifice [51]. In our study, the MTT assay showed that the toxicity of AFCs is completely concentration dependent. These results are in concordance with other research where the inhibition was increased with increased concentration of antimicrobials [52,53].

The GC-MS profiling of the metabolites have been proved to possess antifungal properties that were abundantly present and the antifungal nature of the culture filtrates may be attributed to this. Various scientific literatures have also been correlated with the results obtained in our study [54,55].

The volatile bioactive metabolites responsible for the antagonistic nature of *A. giganteus* was identified by GC-MS profiling. These identified fatty acids and methylated esters along with the non-volatile metabolites may be attributed to the antagonistic potential of *A. giganteus*.

## Conclusion

The application of antimicrobial compounds from natural sources, especially microorganisms has become a great tool for the production and discovery of novel drugs for the treatment and prevention of fungal infections. The interaction studies proved that the sarcin is an effective ligand to inhibit the *A. fumigatus* to manage the infection, aspergillosis in human. The present study strongly revealed the role of AFPs of *A. giganteus* reiterating its antagonistic nature for treating the pathogen causing aspergillosis. The structural and morphological changes in the AFCs treated *A. fumigatus* have revealed that the potential application of AFCs of *A. giganteus* for the effective inhibition. Consecutively, the present work exploits the mechanism of AFCs from *A. giganteus* on the cell wall and cell membrane of *A. fumigatus*, aspergillosis causing pathogen. An irreversible gross morphological change in the treated pathogenic strain was noted, thus causing the release of cellular and cytoplasmic constituents. Moreover, the cytotoxicity level of AFCs of *A. giganteus* proved the safer level of AFCs. The GC-MS profiling of bioactive metabolites in the *A. giganteus* further explains the nature of bioactive metabolites.

## Supplementary Material

Supplementary Files S1-S2

## Data Availability

Data associated with this paper can be accessed by contacting the corresponding author.

## Abbreviations

ADMET: absorption distribution metabolism excretion toxicity profile
AFC: antifungal compound
AFP: antifungal protein
AIDS: acute immunodeficiency syndrome
ANOVA: analysis of variance
CYE: Czapek yeast extract medium
DNA: deoxyribonucleic acid
FE-SEM: field emission scanning electron microscope
GC-MS: gas chromatography–mass spectrometry
MTT: 3-(4,5-dimethylthiazol-2-yl)-2,5-diphenyl-2H-tetrazolium bromide
NIST: National Institute of Standards and Technology
OPLS: optimized potentials for liquid simulations
PDB: Protein Data Bank
RMSD: root-mean-square deviation
RNA: ribonucleic acid
