# Supplementary material for: Pharmacokinetic profile of sarcin and thionin from Aspergillus giganteus and in vitro validation against human fungal pathogen
Source: Biosci Rep. 2022 Sep 7;42(9):BSR20220229. doi: 10.1042/BSR20220229 (PMC9469106; doi:10.1042/BSR20220229)
Supplement: Supplementary Files S1-S2 [file BSR-2022-0229_supp.pdf]

Supplementary file 1: Profile description of sarcin, thionin, chitinase and its derivatives of *Aspergillus giganteus*

Selected derivatives	Compound	PubChem ID	Molecular Weight	Log P	Rotatable bonds	Acceptors	Donors	Surface area
Standard Drug	Fluconazole	3365	306.276	0.7358	5	7	1	123.419
Sarcin	1-Methylthioguianine	3032391	181.224	0.60809	0	5	2	73.255
	3-Imino-3H-phenothiazin-7-amine	65044	227.292	2.46277	0	4	2	96.045
Thionin	Thionine cation	462371	228.3	0.6431	0	3	2	96.045
	3-Methyl-2-[[(8E)-7-methylidene-5-(trifluoromethyl)-3,4,5,6-tetrahydro-2H-thionin-8-yl]methyl]-7-(4-methylimidazol-1-yl)-3,4-dihydropyrido[1,2-a]pyrazine-1,6-dione	122678533	492.567	4.72242	3	6	0	199.708
	N,N-Dimethyl-4-[(Z)-[(3Z,6Z,8Z)-2H-thionin-5-ylidene]methyl]aniline	88094842	269.413	4.5089	2	2	0	119.564
Chitinase	Chitinase-IN-2	86223064	395.488	2.45152	6	7	1	166.525
	Chitinase-IN-1	86223063	352.419	2.38552	5	6	1	148.035
	Chitin from Shrimp cells	6857375	221.209	-3.0776	2	6	5	86.290

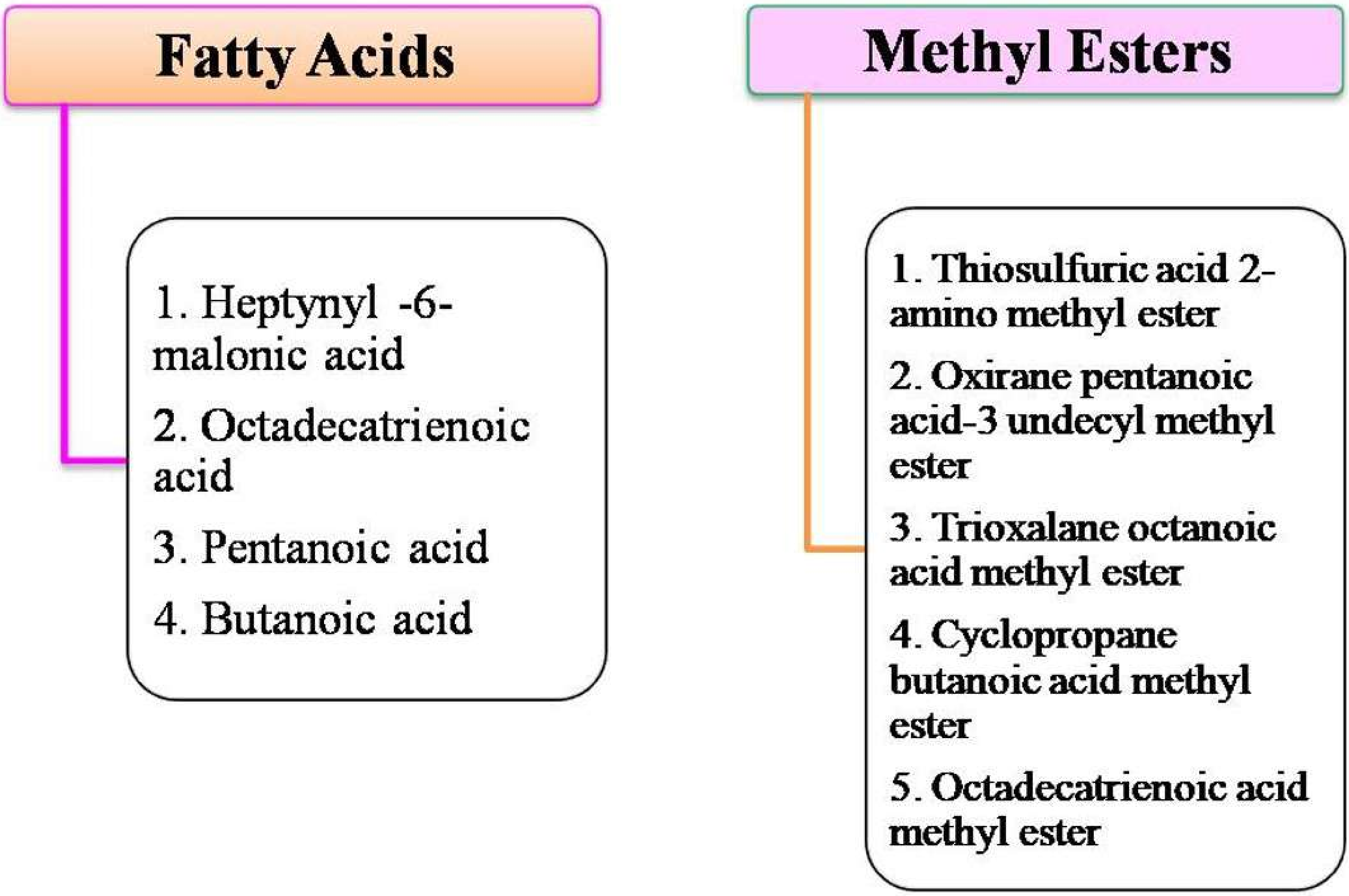

Supplementary file 2. Fatty acids and methyl esters identified in GC-MS of bioactive compounds of *Aspergillus giganteus*
